# An Oncolytic Recombinant Vesicular Stomatitis Virus Expressing mGM-CSF and mIL-12 Enhances Antitumour Efficacy in a U-87 MG Glioblastoma Xenograft Model

**DOI:** 10.3390/cancers18142291

**Published:** 2026-07-16

**Authors:** Nizami B. Gasanov, Vasiliy Moroz, Dmitriy Ovcharenko, Mariia Toropko, Roman Ivanov, Alexander Karabelsky

**Affiliations:** 1 Research Center for Translational Medicine, Sirius University of Science and Technology, Sirius Federal Territory, 1 Olympic Ave., Sirius 354340, Russia; moroz.vd@talantiuspeh.ru (V.M.); ivanov.ra@talantiuspeh.ru (R.I.); karabelskiy.av@talantiuspeh.ru (A.K.); 2 Altogen Labs, 11200 Menchaca Road, Austin, TX 78748, USA

**Keywords:** vesicular stomatitis virus, VSV, rVSV-dM51-mIL12-mGMCSF, Ki-67 H-score, glioblastoma model, glioblastoma therapy, intratumoural transgene expression, U-87 MG xenograft model, oncolytic

## Abstract

**Background**: Glioblastoma (GBM) is characterised by therapeutic resistance and high invasiveness, so the development of new treatments is essential. Oncolytic virotherapy using the vesicular stomatitis virus (VSV) is a promising approach as it is inherently tumour-selective and immunostimulatory. This study evaluated the antitumour efficacy of a recombinant VSV engineered to co-express mouse interleukin-12 and granulocyte-macrophage colony-stimulating factor (rVSV-dM51-mIL12-mGMCSF) using in vitro and in vivo U-87 MG models. **Methods**: The oncolytic activity of rVSV-dM51-mIL12-mGMCSF was evaluated in vitro against human U-87 MG and mouse GL-261 glioblastoma cells using flow cytometry (MOI 0.1) and MTT assays (MOIs 0.1 and 0.001). In vivo, tumour progression was monitored in U-87 MG xenograft mice for 30 days after inoculation. At the study endpoint, tumour tissues from treated and control animals were subjected to immunohistochemical (IHC) analysis; the H-score method was used to quantify expression of the Ki-67 proliferation marker, VSV glycoprotein (VSV-G), and mIL-12. **Results**: rVSV-dM51-mIL12-mGMCSF demonstrated oncolytic activity against both cell lines in vitro; however, its cytotoxicity was lower compared to the parental control virus (rVSV-dM51-GFP). In contrast, treatment in vivo resulted in greater tumour growth inhibition. IHC analysis revealed a significant reduction in the Ki-67 H-score alongside high levels of VSV glycoprotein and mIL-12 expression. These results confirm that, although the payloads do not enhance direct viral oncolysis in vitro, they significantly improve antitumour efficacy in vivo. **Conclusions**: rVSV-dM51-mIL12-mGMCSF effectively inhibited tumour growth in the U-87 MG xenograft model, supporting further evaluation of glioblastoma-directed oncolytic virotherapy.

## Simple Summary

This study investigates a new immunotherapy approach for glioblastoma, which is one of the most aggressive types of brain cancer. Using the U-87 MG xenograft glioblastoma model, we evaluated the antitumour efficacy of a recombinant vesicular stomatitis virus that co-expresses mouse interleukin-12 and granulocyte-macrophage colony-stimulating factor (rVSV-dM51-mIL12-mGMCSF). Having previously demonstrated the activity of this virus in B16 melanoma and LL2 lung carcinoma models, we investigated its potential to overcome the therapeutic challenges associated with glioblastoma. Our results indicate that rVSV-dM51-mIL12-mGMCSF exhibits similar oncolytic activity to the parental control virus (rVSV-dM51-GFP) in vitro but demonstrates significantly greater efficacy against glioblastoma in vivo. This was associated with delayed tumour growth and reduced Ki-67 expression.

## 1. Introduction

Oncolytic viruses (OVs) are an emerging class of anticancer therapeutics. Since the mid-20th century, extensive studies have investigated the oncolytic potential of various viruses [1,2]. One key advantage of OVs is their genetic plasticity, which enables them to replicate more selectively within malignant cells and lyse them while leaving healthy cells unharmed. OVs can also be genetically modified to target specific molecules on the surface of malignant cells. Specific mutations can restrict viral replication in normal cells, and essential viral genes can be placed under the control of tumour-specific promoters to further enhance safety [3]. OVs can carry various payloads, including pro-apoptotic and immunomodulatory molecules; regulators of metabolism and neovascularisation; prodrugs; and gene expression modulators, such as microRNA (miRNA) precursors and inhibitors [4,5,6].

Several oncolytic virus (OV) drugs have been approved by regulatory authorities worldwide. These include Rigvir^®^, a modified ECHO-7 virus registered in Latvia in 2004 [7,8], and H101 (Oncorine), a p53-deficient targeting adenovirus approved in China in 2005 for use in treating nasopharyngeal and head and neck carcinomas [9,10,11,12,13]. In 2015, the US Food and Drug Administration (FDA) approved talimogene laherparepvec (T-VEC, Imlygic^®^), a herpes simplex virus 1 (HSV-1)-based vector encoding human granulocyte-macrophage colony-stimulating factor (GM-CSF), for treating advanced melanoma [14,15,16,17]. Subsequently, in 2021, Japan approved the third-generation oncolytic HSV-1, Teserpaturev/G47Δ (Delytact^®^), for treating malignant glioma [18,19]. Most recently, in 2022, the FDA approved nadofaragene firadenovec (Adstiladrin^®^, nadofaragene firadenovec-vncg), a non-replicating adenoviral vector that delivers the interferon (IFN)-α2b gene, for treating Bacillus Calmette-Guérin (BCG)-refractory non-muscle-invasive bladder cancer [20,21]. Additionally, novel candidates such as the vaccinia virus CLD-201, which is delivered via mesenchymal stem cells, are currently undergoing expedited regulatory review for various solid tumours [22,23]. OVs include both naturally occurring wild-type viral strains and genetically engineered viruses that selectively replicate in and destroy malignant cells while leaving normal tissues unharmed [2,3]. VSV has been studied as an oncolytic agent due to its rapid life cycle, sensitivity to the type I interferon response and other advantages [24,25]. These characteristics enable VSV to propagate preferentially in malignant cells while remaining non-pathogenic to humans [24,26,27]. Genetic modifications, including mutations in the matrix (M) gene, truncations or deletions in the glycoprotein (G) gene, and insertion of foreign genes, have further attenuated VSV in IFN-competent normal cells while preserving its oncolytic activity in malignant cells, where these pathways are often dysfunctional [28,29,30,31,32]. Numerous studies have demonstrated that the oncolytic activity of VSV can be enhanced through pseudotyping, directed evolution, or the encoding of tumour suppressors, metabolic and epigenetic modulators, apoptosis inducers, and anti-angiogenic or immunomodulatory molecules [33]. Previously, VSV variants engineered to express IFN-β, IFN-γ, CXCL9 and other immune-stimulating molecules have been investigated [32,34,35,36,37].

OVs not only destroy malignant cells, but also release tumour-associated antigens, which stimulate antitumour immune responses. This mechanism is particularly important for immunologically ‘cold’ tumours, which are characterised by an immunosuppressive microenvironment that inhibits the infiltration and activity of immune cells due to the presence of suppressive myeloid and stromal cells [38].

Immunostimulatory proteins delivered by OVs can enhance the antitumour effect. Previous studies have demonstrated that rVSVs loaded with immunostimulatory molecules such as interleukin-12 (IL-12) and granulocyte-macrophage colony-stimulating factor (GM-CSF) can reduce tumour growth and increase the survival rate of C57BL/6 mice subcutaneously implanted with B16-F10 melanoma cells [39]. The rVSV have also been shown to partially inhibit tumour growth in a C57BL/6 murine lung carcinoma model established by subcutaneous injection of LL/2 cells [40].

Glioblastoma multiforme (GBM) is a highly malignant grade IV glioma characterised by a “cold” immunosuppressive tumour microenvironment (TME) and high cellular heterogeneity. The GBM TMEs are characterised by low pH levels, inhibitory cytokines, and a dense accumulation of myeloid-derived suppressor cells, regulatory T cells, and M2-polarised tumour-associated macrophages. These factors severely restrict the efficacy of current immunotherapies, including checkpoint inhibitors, vaccines, and CAR T-cell therapy [41,42,43]. GBM has an extremely poor prognosis, with a median overall survival of less than 15 months and high recurrence rates [44,45]. Despite standard therapeutic strategies combining surgical resection, radiotherapy and temozolomide (TMZ) chemotherapy, these conventional interventions remain insufficient to overcome the disease’s highly aggressive nature [46,47,48].

Previous studies have shown that delivering IL-12 in GBM can promote antitumour immunity by altering the cellular composition of the TME, as well as improving the response to checkpoint blockade therapy [49,50]. Meanwhile, GM-CSF promotes the recruitment of antigen-presenting cells into the TME, enhances dendritic cell function and stimulates cytotoxic T-cell responses to tumour-associated antigens [15]. In a study by Yang et al., the combination of GM-CSF with TMZ and radiotherapy for GBM treatment was found to increase median patient survival [51]. Furthermore, a clinical trial evaluated the use of GM-CSF alongside the IMA950 vaccine against GBM as an immunostimulatory adjuvant [52].

Oncolytic viruses have proven to be a potent delivery platform for IL-12 and GM-CSF, offering reduced systemic toxicity, targeted drug delivery with accumulation at the disease site, and induction of antitumour immunity [53]. In this study, we investigated the oncolytic properties of rVSV encoding murine IL-12 and GM-CSF (rVSV-dM51-mIL12-mGMCSF) by comparing it with the parental control virus expressing green fluorescent protein (rVSV-dM51-GFP) in vitro and in athymic nude mice in vivo, in the context of GBM. Both rVSVs contain a mutation in the matrix gene (ΔM51), which enhances their sensitivity to the interferon (IFN) response [54]. However, rVSV-dM51-mIL12-mGMCSF exhibited reduced potency in vitro, likely due to its larger genome size and consequently lower replication rate. Nevertheless, in vivo studies showed stronger antitumour activity compared with rVSV-dM51-GFP.

## 2. Materials and Methods

### 2.1. Cell Culture

Human glioblastoma U-87 MG cells (HTB-14, ATCC), mouse glioblastoma GL-261 cells (ACC 802, DSMZ), and BHK-21 cells (85011433, ECACC) were obtained from the American Type Culture Collection (ATCC), the German Collection of Microorganisms and Cell Cultures (DSMZ), and the European Collection of Authenticated Cell Cultures (ECACC), respectively. All cell lines were cultured in Dulbecco’s Modified Eagle’s Medium (DMEM) containing 4.5 g/L glucose, supplemented with 10% foetal bovine serum (FBS), at 37 °C in a humidified atmosphere containing 5% CO_2_.

### 2.2. Construction of Plasmids

**Figure 1:**
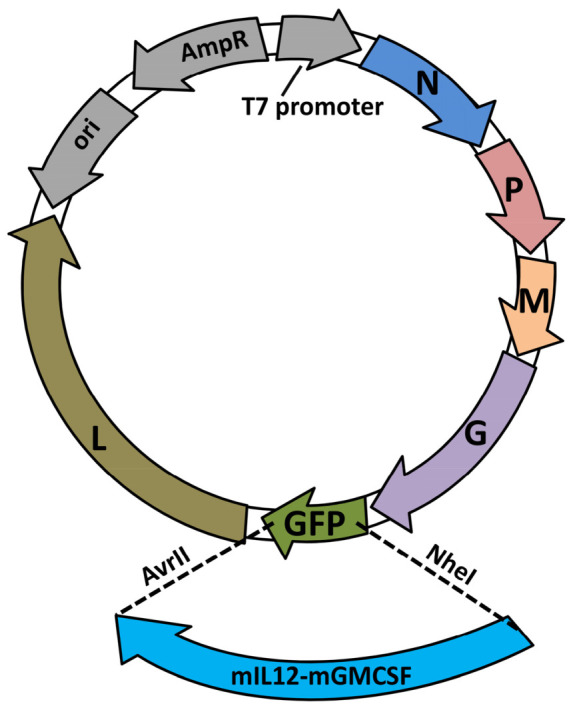
Schematic representation of the pVSV-dM51-GFP plasmid vector. This circular genomic map shows the organisation of the viral coding sequences (N, P, M, G and L genes), the therapeutic mIL12–mGMCSF fusion cassette and the unique NheI and AvrII restriction sites used for cloning.

All plasmids were generated using a methodology that we had previously developed [39,40,55]. The pVSV-dG-GFP plasmid (#EH1003, Kerafast, Boston, MA, USA) was used as the backbone for creating the recombinant VSV constructs. Two plasmids, pVSV-dM51-GFP and pVSV-dM51-mIL12-mGMCSF, were engineered to encode the five canonical VSV proteins alongside a green fluorescent protein (GFP) reporter, with the mIL12-mGMCSF fusion (2052 bp) replacing the GFP gene. The mIL12-mGMCSF fusion cassette was cloned into the pVSV-dM51-GFP plasmid vector, replacing the GFP gene located in the intergenic region between the G and L viral genes, using the NheI and AvrII restriction sites [39] (Figure 1).

These plasmids encode a matrix (M) protein with a deletion of the methionine codon at position 51 (ΔM51). To rescue infectious rVSV particles, plasmid pCAGT7pol (#59926, Addgene, Watertown, MA, USA), encoding T7 RNA polymerase, was utilised. This plasmid was also used as the backbone for constructing helper plasmids (pCAG-N, pCAG-P and pCAG-L) designed to express the VSV nucleocapsid (N), phosphoprotein (P) and large polymerase (L) proteins under the transcriptional control of the CAG promoter. The integrity of all plasmid sequences was confirmed by DNA sequencing.

### 2.3. Rescue and Amplification of Recombinant VSVs

The rescue of replication-competent rVSV-dM51-GFP and rVSV-dM51-mIL12-mGMCSF was performed using a helper virus-free method, as previously described [55], with two modifications: (1) the use of BHK-21 cells, and (2) the delivery of T7 RNA polymerase via transfection with the pCAG-T7pol plasmid. BHK-21 cells were grown overnight in a 12-well plate until they reached 80–85% confluency. The following day, the cells were co-transfected with a mixture of plasmids (pCAG-VSVN, pCAG-VSVP, pCAG-VSVL, pCAGGS-G, pCAG-T7pol and either pVSV-dM51-GFP or pVSV-dM51-mIL12-mGMCSF) at mass ratios of 3:5:1:4:5:8, respectively. Polyethyleneimine was used as the transfection reagent at a ratio of 5:1 to the total DNA (13.0 µg). After 16 h, the medium was replaced with DMEM supplemented with 5% FBS and the cells cultured at 37 °C in a 5% CO_2_ incubator. The cell culture supernatant was harvested 72 h post-transfection and used for serial virus amplification. For the initial amplification, BHK-21 cells were grown in 12-well plates until they reached 80–85% confluency, after which they were inoculated with 500 µL of the supernatant containing the rescued rVSV-dM51-GFP or rVSV-dM51-mIL12-mGMCSF virus. One and a half hours later, 500 µL of fresh DMEM containing 4% FBS and 2 mM L-glutamine was added. GFP expression and the cytopathic effect (CPE) were detected using an inverted fluorescence microscope (Carl Zeiss Microscopy GmbH, Jena, Germany) 24 h later. The supernatant was collected after 72 h, then centrifuged at 3000× *g* for 10 min at 4 °C. To generate high-titre, large-volume viral stocks, BHK-21 cells at 70–80% confluency in T-25 flasks were inoculated at an MOI of 10^−4^ with rVSV supernatant from previous productions. The cells were maintained in DMEM supplemented with 2% FBS and 2 mM L-glutamine, and incubated for 72 h. Following amplification in T-25 flasks, the viral supernatants were collected and centrifuged at 3000× *g*. They were then filtered through a 0.45 µm membrane. The clarified viral preparations (rVSV) were stored at either 4 °C or −80 °C, or used immediately for further amplification, purification or analysis. The titres of the rVSV preparations were determined to be 50% tissue culture infectious doses (TCID_50_)/mL by assessing CPE in BHK-21 cells 72 h post-infection (hpi), calculated using the Reed–Muench method [55,56].

### 2.4. Flow Cytometry

Cell viability was assessed by flow cytometry at 24, 48 and 72 hpi. The cells were harvested by trypsinisation and resuspended in 225 µL of FACS buffer (1× PBS and 3% FBS). Methyl green was then added at a dilution of 1:20,000 as a viability dye/indicator. Data were acquired using a gating strategy focused on single, viable cells on a CytoFLEX flow cytometer (model No. A00-1-1102; Beckman Coulter, Inc., Brea, CA, USA) with CytExpert software (version 2.4.0.28).

### 2.5. MTT Assay

The cytotoxicities of rVSV-dM51-GFP and rVSV-dM51-mIL12-mGMCSF were assessed using the MTT assay (Mosmann, 1983) [57]. An MTT Cell Growth Assay Kit (#CT02, Sigma-Aldrich, St. Louis, MO, USA) was used for this purpose, according to the manufacturer’s instructions. Briefly, U-87 MG cells were seeded at a density of 50,000 cells per well in 96-well plates. The cells were then cultured overnight in DMEM supplemented with 10% FBS at 37 °C in an atmosphere containing 5% CO_2_. The following day, the cell monolayers were treated with rVSV-dM51-GFP and rVSV-dM51-mIL12-mGMCSF at MOIs of 0.1 and 0.001, after which the culture medium was replaced with a medium containing 2.2% FBS. This low serum concentration was empirically optimised to maintain cell viability while preventing overgrowth during the experiment. After 48 and 72 h, the absorbance was measured at 570 and 630 nm using a ClarioStar microplate reader (BMG Labtech, Ortenberg, Germany). Cell viability was calculated relative to the control groups (uninfected cells maintained under identical culture conditions).

### 2.6. Animal Studies

Ten- to 12-week-old female Mus musculus NU(NCr)-Foxn1nu mice were purchased from Inotiv (Livermore, CA, USA). The mice underwent a mandatory one-week acclimatisation period. They were housed in individual cages of up to five mice per cage under the following conditions: a temperature of 22–25 °C, humidity of 40–60%, a 12 h light/dark cycle, irradiated sterile individually ventilated cages, irradiated corncob bedding, and an immunocompromised mouse diet that included irradiated sterilised dry granule food and sterile autoclaved water. Animal procedures and maintenance followed institutional guidelines. The mice were housed at the Altogen Labs animal facility. Phosphate-buffered saline was obtained from Bio-Rad (#1610780; Hercules, CA, USA), Matrigel from Corning Life Sciences (#354248; Tewksbury, MA, USA), and other reagents from Thermo Fisher Scientific (Waltham, MA, USA) unless otherwise specified.

### 2.7. Tumour Inoculation

The U-87 MG cell line was cultured in DMEM supplemented with 10% FBS. The cells were maintained in Corning T-75 flasks in a humidified atmosphere at 37 °C with 5% CO_2_. The cells were passaged when they reached 80–90% confluency. For tumour inoculation, cells in the exponential growth phase were harvested. A total of 50 × 10^6^ viable U-87 MG cells were prepared for xenotransplantation and analysed for cell count and viability (>99.2%) using a GUAVA PCA flow cytometer (Sigma-Aldrich). Each mouse was inoculated subcutaneously at the flank with 1 × 10^6^ U-87 MG cells in 0.1 mL of 1× PBS mixed with Matrigel at a ratio of 1:1. Tumours were allowed to grow until they reached a measurable size of 50–100 mm^3^, which occurred 96 h post-xenotransplantation [58]. Twenty-one animals bearing tumours within this size range were then selected for subsequent experiments.

### 2.8. Study Design and Randomization

Tumour cell inoculation was designated as day 0. On day 5, the viral preparations were administered. Prior to the start of treatment, all mice were weighed, and tumour volumes were measured using digital callipers to confirm sizes of 50–100 mm^3^. To ensure a uniform tumour burden across groups, the mice were assigned using a randomised block design. The animals were first stratified into blocks based on their initial tumour volume. Within each block, the experimental animals were randomly allocated to one of three treatment groups (*n* = 7 mice per group): Group 1 (rVSV-dM51-mIL12-mGMCSF), Group 2 (parental control rVSV-dM51-GFP) and Group 3 (placebo control, DMEM vehicle). This approach ensured balanced baseline tumour volumes across groups. To standardise the treatment dose at 1 × 10^7^ infectious particles per administration across the viral groups, the injection protocols were adjusted based on stock titres. Due to the lower stock titer, Group 1 received three sequential 20 µL intratumoural (IT) injections (60 µL in total) on each treatment day. For Group 2, the higher-titre virus was diluted to deliver an identical dose of 1 × 10^7^ infectious particles in a single 20 µL IT injection. Group 3 received a single 20 µL IT injection of DMEM.

### 2.9. Tumour Growth Inhibition Analysis

Tumour growth inhibition was quantified by calculating the tumour growth inhibition index (TGII) as a percentage (TGII%). First, the relative tumour volume (RTV) was determined for each group by dividing the average tumour volume on a given measurement day by the average tumour volume on day 0. The TGII% was then calculated using the following formula:

TGII% = [1 − (RTV of treated group/RTV of control group)] × 100.

### 2.10. Immunohistochemical Analysis

Upon termination of the in vivo study, tumour-bearing animals were humanely euthanised via CO_2_ asphyxiation, followed by cervical dislocation. Tumour tissues were aseptically excised from two experimental groups: the control group (DMEM Vehicle Control) and the treatment group (rVSV-dM51-mIL12-mGMCSF). The tissues were then immediately fixed in 10% neutral buffered formalin (NBF) from Globe Scientific (#6522FL; Mahwah, NJ, USA) for 48 h at room temperature. To minimise cold ischaemic time, tissues were placed in 10% NBF within 5 min of excision. A 10:1 formalin-to-tissue volume ratio was used for fixation. The NBF solution was freshly prepared and pH-stabilised (7.0 ± 0.2) to ensure optimal tissue preservation and antigen stability. After 48 h at room temperature, the tissues were dehydrated using a graded ethanol-xylene series, embedded in paraffin and sectioned at 4 µm using a rotary microtome. The resulting sections were mounted onto positively charged glass slides (Apex^®^ Superplus Adhesive Slides, Leica Microsystems, Buffalo Grove, IL, USA) and air-dried overnight. The formalin-fixed, paraffin-embedded (FFPE) tissue sections were then subjected to immunohistochemical (IHC) staining using a standardised protocol on a Leica Bond RX automated stainer from Leica Microsystems. All steps were performed in accordance with validated standard operating procedures, with slides processed in parallel to minimise inter-batch variability. The sections were initially deparaffinised and heat-induced epitope retrieval (HIER) was performed using a citrate-based buffer solution (pH 6.0) from Leica Microsystems (#AR9961; Buffalo Grove, IL, USA) for 20 min. After deparaffinisation and epitope retrieval in validated pH-dependent buffers, the slides were blocked for endogenous peroxidase activity and non-specific protein binding using Peroxidazed 1 (#PX968) and Sniper Blocking Reagent (#BS966), both from Biocare Medical (Pacheco, CA, USA), prior to incubation with the primary antibody. The sections were then incubated with the following primary antibodies: anti-Ki67 from Abcam (#ab16667, Cambridge, UK) at a dilution of 1:500 for 60 min; anti-VSV-G from Santa Cruz Biotechnology (#SC-365019, Dallas, TX, USA) at a dilution of 1:2000 for 30 min; and anti-IL-12 from Abcam (#ab131039, Cambridge, UK) at a dilution of 1:800 for 30 min. The antibody dilutions were empirically optimised, and the specificity was validated using positive and negative control tissues, including secondary antibody-only controls to confirm the absence of non-specific binding. Antigen–antibody complexes were detected using the Bond Polymer Refine Detection Kit from Leica Microsystems (#DS9800; Buffalo Grove, IL, USA), which employs a diaminobenzidine chromogen for visualisation. Hematoxylin was used as a nuclear counterstain. The stained slides were then dehydrated, cleared and mounted using a Tissue-Tek Prisma coverslipper system from Sakura Finetek (Torrance, CA, USA). Protein expression was quantified using a semi-quantitative H-score. The H-score was calculated by assessing the percentage of tumour cells exhibiting absent (0), weak (1+), moderate (2+), or strong (3+) staining intensity, using the formula: H-score = [(% 1+ cells × 1) + (% 2+ cells × 2) + (% 3+ cells × 3)], providing a theoretical range of 0 to 300.

### 2.11. Statistical Analysis

The tumour volume progression data from the in vivo experiment were analysed using an ordinary one-way ANOVA test with a Tukey’s multiple comparisons test. The statistical analysis of the IHC data from the in vivo experiments was performed using an unpaired *t*-test. The cell viability data from the in vitro experiments were analysed using multiple unpaired two-tailed *t*-tests with the Benjamini, Krieger and Yekutieli two-stage step-up procedure. All statistical analyses were carried out using GraphPad Prism 8.2.1 software. A *p*-value of <0.05 (*) was considered statistically significant, as were *p*-values of <0.01 (**), <0.001 (***) and <0.0001 (****). A *p*-value of >0.05 (ns) was considered not significant.

## 3. Results

### 3.1. rVSV-dM51-mIL12-mGMCSF Suppressed Tumour Growth in the U-87 MG Xenograft Model

Previous studies in B16-F10 melanoma and Lewis lung carcinoma models demonstrated that administration of rVSV-dM51-mIL12-mGMCSF resulted in significant tumour growth regression [39,40]. The present study assessed the therapeutic efficacy of the recombinant oncolytic virus in a U-87 MG xenograft mouse model of malignant glioma. To establish this model, immunodeficient NU(NCr)-Foxn1nu (athymic nude) mice were inoculated subcutaneously with 1 × 10^6^ U-87 MG cells [58].

**Figure 2:**
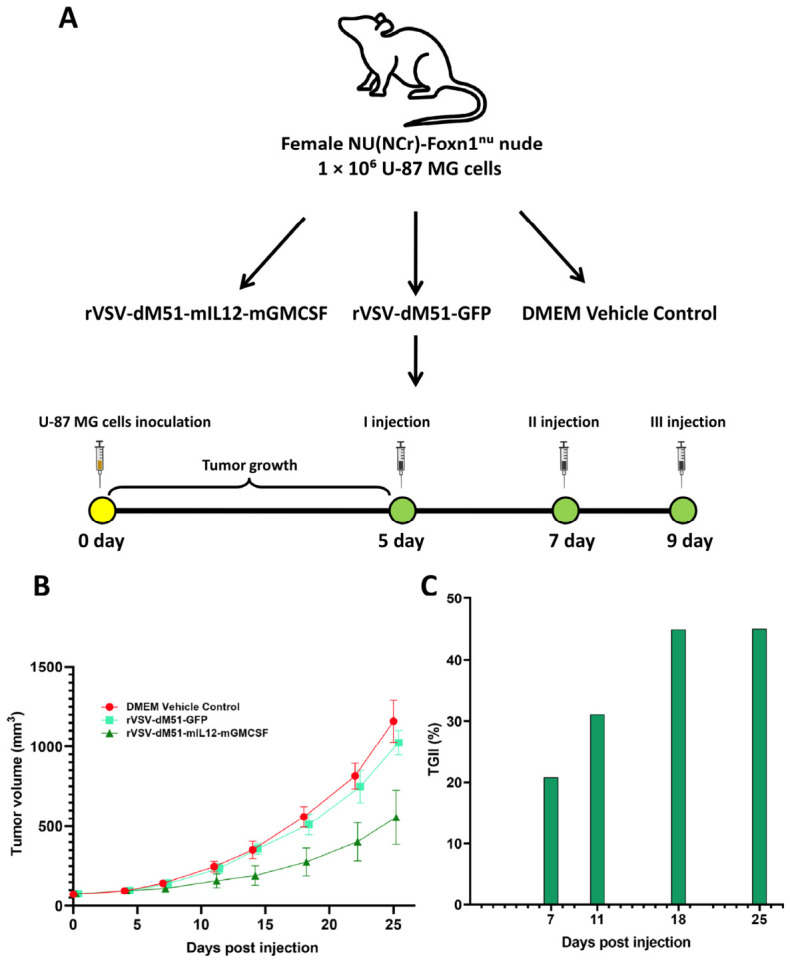
Antitumour efficacy of rVSV-dM51-mIL12-mGMCSF in the U-87 MG xenograft model. (**A**) Schematic representation of the experimental design and treatment schedule. (**B**) Tumour volume progression (mm^3^) over time across the three treatment groups. The rVSV-dM51-mIL12-mGMCSF group exhibited significant tumour growth inhibition compared to the control groups. (**C**) The tumour growth inhibition index (TGII, %): rVSV-dM51-mIL12-mGMCSF relative to rVSV-dM51-GFP, demonstrating the enhanced therapeutic potency of the recombinant virus co-expressing mIL-12 and mGM-CSF.

Treatment with rVSV commenced on day 5 post-inoculation when tumour volumes reached 50–100 mm^3^. On days 5, 7 and 9 post-tumour inoculation, the mice received IT injections of either rVSV-dM51-mIL12-mGMCSF (Group 1), rVSV-dM51-GFP (parental control virus, Group 2), or DMEM only (Group 3) (Figure 2A). All viral administrations were strictly standardised to deliver an equivalent dose of 1 × 10^7^ infectious particles per mouse on each treatment day, as detailed in Section 2.8. Tumour growth was monitored by measuring tumour size every 3–4 days for 30 days. All animals survived the 30-day study period, and no treatment-related mortality was observed. Daily clinical observations revealed no signs of toxicity, abnormal behaviour or body weight loss exceeding 20% of baseline levels. General physical parameters, including grooming behaviour, posture and mobility, remained within normal ranges across all cohorts. Treatment with rVSV-dM51-mIL12-mGMCSF (Group 1) resulted in statistically significant tumour growth inhibition compared to the placebo group (Group 3). The therapeutic effect was first observed on day 7 post-injection (*p* < 0.05 vs. Group 3), becoming more pronounced from day 11 onwards. Significance was maintained throughout the study until the endpoint on day 25 (*p* < 0.0001) (Figure 2B). In contrast, the control virus rVSV-dM51-GFP (Group 2) showed no significant antitumour activity compared to the placebo group (Group 3). Significant differences in tumour volumes were also observed between groups 1 and 2, commencing on day 11 post-injection (*p* < 0.05) and persisting until the study endpoint on day 25 (*p* < 0.0001). These data suggest that rVSV-dM51-mIL12-mGM-CSF was the most effective treatment for suppressing U-87 MG tumour progression (Figure 2B). Partial inhibition of tumour growth was observed in Group 1 compared with Groups 2 and 3 throughout the 25-day post-injection period. The tumour growth inhibition index (TGII%) for Group 1, relative to Group 2, reached a maximum of 45.8% on day 14 and remained stable through to day 25 (45.0%; Figure 2C).

### 3.2. Immunohistochemical Analysis Confirmed Intratumoral Transgene Expression and Reduced Tumour Cell Proliferation

Intratumoural viral delivery, transgene expression, and the subsequent biological effects were evaluated by quantitative IHC analysis of U-87 MG xenograft tumours following IT administration of rVSV-dM51-mIL12-mGMCSF. The expression levels of the vesicular stomatitis virus glycoprotein (VSV-G), the therapeutic cytokine IL-12 and the proliferation marker Ki-67 were quantified using the H-score method, which integrates staining intensity and percentage of positive cells.

#### 3.2.1. Intratumoural Expression of VSV-G and IL-12 Following Viral Administration

**Figure 3:**
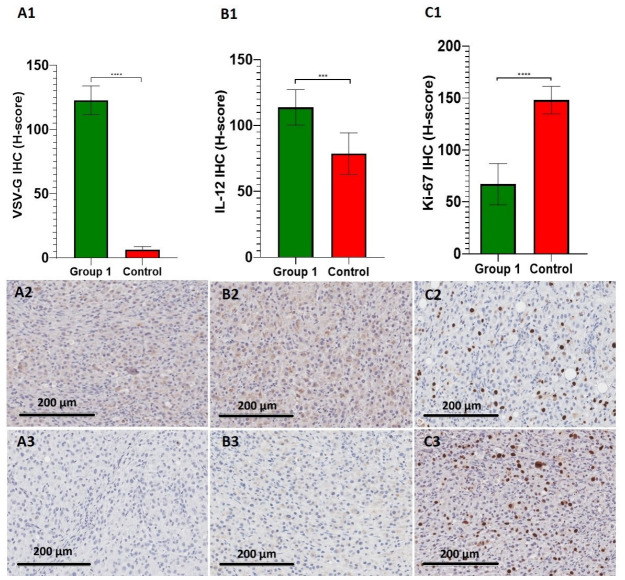
Immunohistochemical analysis and quantification of VSV-G, IL-12 and Ki-67 expression in tumour tissues. (**A1**,**B1**,**C1**) H-score quantification of VSV-G, IL-12 and Ki-67 expression in tumour sections from rVSV-dM51-mIL12-mGMCSF-treated and untreated control mice. (**A2**,**A3**) Representative photomicrographs of VSV-G immunohistochemical staining in (**A2**) rVSV-dM51-mIL12-mGMCSF-treated and (**A3**) untreated tissues. (**B2**,**B3**) Representative photomicrographs of IL-12 immunohistochemical staining in (**B2**) rVSV-dM51-mIL12-mGMCSF-treated and (**B3**) untreated tissues. (**C2**,**C3**) Representative photomicrographs of Ki-67 immunohistochemical staining in (**C2**) rVSV-dM51-mIL12-mGMCSF-treated and (**C3**) untreated tissues. Scale bars = 200 μm; magnification: 20×. *** *p* <0.001, **** *p* < 0.0001.

To confirm viral infection and IL-12 transgene expression in vivo, quantitative (IHC) analysis using the H-score method was performed on tumour tissues harvested from mice following an IT injection of either rVSV-dM51-mIL12-mGMCSF (Group 1) or DMEM (Control). The tumours were harvested 25 days after administration of the virus or DMEM intratumourally. Representative IHC photomicrographs revealed distinct differences between the two groups. While tumours from DMEM-treated control mice exhibited only background, non-specific staining for both VSV-G and IL-12 (Figure 3(A3,B3)), sections from tumours treated with rVSV-dM51-mIL12-mGMCSF showed abundant and distinct positive staining (Figure 3(A2,B2)). For quantitative assessment, the H-score (ranging from 0 to 300) was calculated for each marker. To ensure representative sampling and account for tumour heterogeneity, a minimum of eight randomly selected tumour-rich regions were scored per slide. Necrotic, stromal and non-tumour tissue compartments were systematically excluded from the analysis to restrict quantification to viable tumour cells. The final H-score assessment was derived from a comprehensive review of multiple fields. Statistical analysis confirmed a highly significant increase in both markers within the virus-treated group (Figure 3(A1,B1)). The mean H-score for VSV-G in virus-treated tumours was 122.8 ± 11.2 (mean ± SD), compared to 6.4 ± 2.5 in control tumours (*p* < 0.0001). This confirmed robust intratumoural viral protein expression. Similarly, IL-12 expression was significantly elevated, with a mean H-score of 113.8 ± 13.4 in the virus-treated group, compared to 78.8 ± 15.7 in the control group (*p* < 0.001). Taken together, these data confirm the successful intratumoural delivery, infection and transgene expression of rVSV-dM51-mIL12-mGMCSF, as evidenced by the high and correlated expression of VSV-G and IL-12.

#### 3.2.2. rVSV-dM51-mIL12-mGMCSF Reduces the Ki-67 Proliferation Index in U-87 MG Xenograft Tumours

To quantitatively evaluate the impact of rVSV-dM51-mIL12-mGMCSF on tumour cell proliferation, IHC analysis of the proliferation marker Ki-67 was performed on excised tumours from both the virus-treated and control group mice. The results were quantified using the H-score method (Figure 3(C1–C3)). Representative IHC staining revealed a significant decrease in Ki-67 staining intensity in tumours treated with rVSV-dM51-mIL12-mGMCSF, as opposed to the control group treated with DMEM (Figure 3(C2,C3)). The mean H-score for Ki-67 in virus-treated tumours was 67.2 ± 19.9 (mean ± SD), compared to 148.1 ± 13.2 in the control group (*p* < 0.0001) (Figure 3(C1)). Overall, the histological data demonstrate that administration of rVSV-dM51-mIL12-mGMCSF markedly reduces Ki-67 expression. This reduction in Ki-67 expression is consistent with decreased tumour cell proliferation in treated xenografts.

### 3.3. In Vitro Cytotoxic Activity of rVSV-dM51-mIL12-mGMCSF in Human and Mouse Glioblastoma Cells

To evaluate the in vitro cytotoxicity of rVSV-dM51-mIL12-mGMCSF against human U-87 MG and mouse GL-261 glioblastoma cells, cell viability was assessed post-infection using flow cytometry and MTT assays. The cells were infected with either rVSV-dM51-mIL12-mGMCSF or the rVSV-dM51-GFP control virus, and cell viability was quantified at 24-, 48-, and 72 h intervals.

#### 3.3.1. Cell Viability Analysis by Flow Cytometry

**Figure 4:**
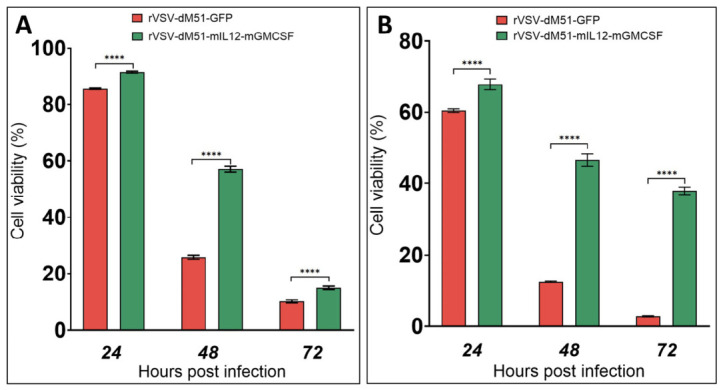
Flow cytometric assessment of glioblastoma cell viability following infection with rVSV-dM51-mIL12-mGMCSF and rVSV-dM51-GFP. (**A**) Human U-87 MG cells, (**B**) mouse GL-261 cells. The data represent the percentage of viable cells at 24, 48, and 72 hpi at an MOI of 0.1 for both viruses. **** *p* < 0.0001.

In order to characterise the cytotoxic activity of rVSV-dM51-mIL12-mGMCSF in vitro, we performed flow cytometric analyses of both human and mouse glioblastoma cells at 24, 48 and 72 hpi with rVSV-dM51-mIL12-mGMCSF and rVSV-dM51-GFP at 0.1 MOI (Figure 4). In human U-87 MG cells (Figure 4A), at 24 h, both viruses induced minimal levels of cell death, with viability remaining above 80%. However, from 48 hpi onwards, a divergence in CPE became apparent. While rVSV-dM51-GFP infection resulted in 26.0 ± 0.7% (mean ± SD) viable cells, rVSV-dM51-mIL12-mGMCSF infection demonstrated significantly weaker CPE, with 57.0 ± 1.0% of cells remaining viable (*p* < 0.0001). This difference in CPE persisted 72 h after infection, with respective percentages of 10.2 ± 0.6% and 15.0 ± 0.6% (*p* < 0.0001). A fully analogous trend was observed in GL-261 mouse glioblastoma cells (Figure 4B). At 24 hpi, cells infected with rVSV-dM51-GFP and rVSV-dM51-mIL12-mGMCSF exhibited viability rates of 60.46 ± 0.49% and 67.84 ± 1.48%, respectively (*p* < 0.0001). By 48 hpi, rVSV-dM51-GFP infection substantially reduced the cell population, leaving only 12.37 ± 0.18% of cells viable, whereas rVSV-dM51-mIL12-mGMCSF exhibited lower cytotoxic activity, with 46.62 ± 1.81% of cells remaining viable (*p* < 0.0001). This delayed cell death remained evident at 72 hpi, with only 2.83 ± 0.19% of cells remaining viable for the parental control virus compared to 37.88 ± 1.04% for the cytokine-expressing vector (*p* < 0.0001). These results show that, despite the marked cytopathic effect of rVSV-dM51-mIL12-mGMCSF on both human and mouse glioblastoma cells in vitro, the insertion of the mIL12-mGMCSF transgene into the viral genome attenuated the cytopathic effect of this virus compared with rVSV-dM51-GFP.

#### 3.3.2. Cell Viability Analysis by MTT Assay

**Figure 5:**
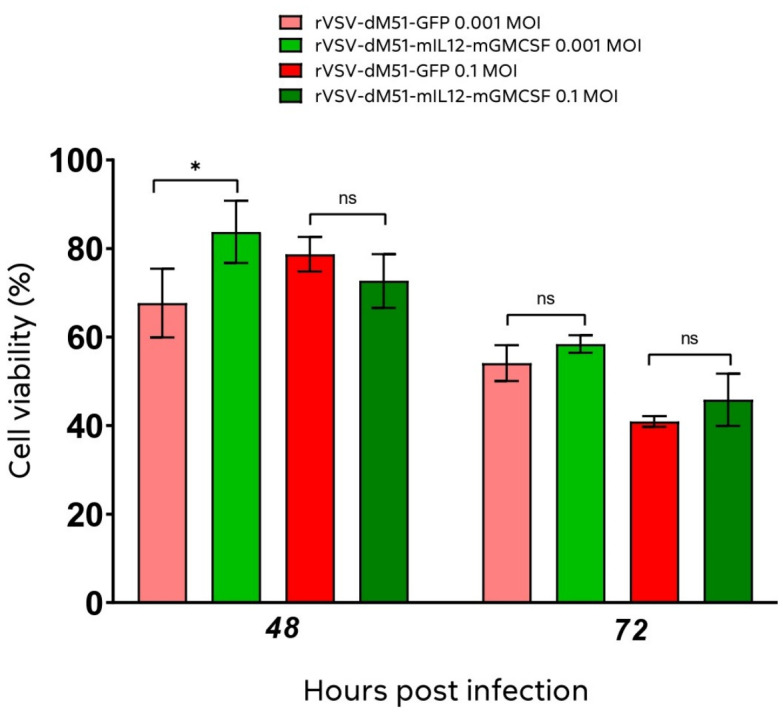
Assessment of U-87 MG cell viability via an MTT assay following infection with rVSV-dM51-mIL12-mGMCSF and rVSV-dM51-GFP. The data show the percentage of viable cells at 48 and 72 hpi with an MOI of 0.001 and 0.1 for both viruses. * *p* < 0.05.

To further evaluate the in vitro cytotoxic activity of rVSV-dM51-mIL12-mGMCSF against U-87 MG cells, MTT assays were performed at MOIs of 0.001 and 0.1. Infection with rVSV-dM51-mIL12-mGMCSF resulted in a significant, time- and dose-dependent reduction in cell viability. However, the cytotoxic effect of rVSV-dM51-mIL12-mGMCSF was not statistically different from that of the control virus, rVSV-dM51-GFP, except in one case (Figure 5). At 48 hpi and an MOI of 0.001, rVSV-dM51-mIL12-mGMCSF reduced U-87 MG viability to 83.8 ± 7.1% (mean ± SD) relative to uninfected controls. This was significantly higher than the viability observed with rVSV-dM51-GFP (67.7 ± 7.7%; *p* < 0.0441). At the higher MOI of 0.1, both viruses reduced cell viability to a similar extent (72.7 ± 6.1% for rVSV-dM51-mIL12-mGMCSF and 78.7 ± 3.9% for rVSV-dM51-GFP), with no significant difference between the two groups (Figure 5; *p* > 0.05). By 72 hpi, cytotoxicity was more pronounced for both viruses. Infection with rVSV-dM51-mIL12-mGMCSF at an MOI of 0.001 resulted in a cell viability of 58.4 ± 2%, which did not differ significantly from that of rVSV-dM51-GFP (54.1 ± 4.1%; *p* > 0.05). Increasing the MOI to 0.1 resulted in a cell viability of 45.8 ± 5.9% for rVSV-dM51-mIL12-mGMCSF compared to 40.9 ± 1.2% for rVSV-dM51-GFP (Figure 5; *p* > 0.05). Therefore, while rVSV-dM51-mIL12-mGMCSF exhibited potent, time- and dose-dependent cytotoxicity against U-87 MG cells in vitro, it was not statistically more effective than rVSV-dM51-GFP under the tested conditions.

## 4. Discussion

The treatment of glioblastoma (GBM) remains a significant challenge in the field of clinical neuro-oncology [59]. GBM exhibits a high level of genetic heterogeneity and a ‘cold’ immunophenotype, which typically makes it resistant to conventional treatments [60,61]. This resistance is driven by a complex immunosuppressive microenvironment that blocks antitumour immunity and limits the efficacy of available therapies [61,62]. In this context, oncolytic virotherapy emerges as a promising strategy for overcoming the aforementioned therapeutic obstacles. The antitumour efficacy of oncolytic viruses is due to their direct lysis of malignant cells, which triggers a stronger local immune response [63]. As demonstrated in our previous research, rVSV-dM51-mIL12-mGMCSF has been shown to be highly potent in melanoma [39] and lung cancer [40] models. In the present study, a U-87 MG glioblastoma model was employed to evaluate the efficacy of this virus against one of the most aggressive and treatment-resistant forms of primary brain cancer. Our results show that rVSV-dM51-mIL12-mGMCSF is also potent in this in vivo glioblastoma model. During the 25-day observation period, sustained inhibition of tumour progression was observed. The tumour growth inhibition index (TGII) for rVSV-dM51-mIL12-mGMCSF relative to the parental control virus (rVSV-dM51-GFP) reached a maximum of 45.8% on day 14, remaining stable until day 25 post-injection. Quantitative immunohistochemistry supported this observation; high H-scores for VSV-G and mIL-12 indicate that the ΔM51 mutation did not prevent viral entry or replication within the U-87 MG tumour mass. Of particular significance was the marked reduction in the Ki-67 H-score (from 148.1 to 67.2), which provides clear evidence of the therapeutic effect on malignant cell proliferation. In order to clarify the mechanisms of this effect in athymic nude mice, a model characterised by a defective adaptive immune system, it was necessary to determine whether the effect was due to the activation of residual innate immunity or direct viral oncolysis. Consequently, the viability of human U-87 MG and mouse GL-261 glioblastoma cells was evaluated in vitro following infection with either rVSV-dM51-mIL12-mGMCSF or rVSV-dM51-GFP. To ensure a comprehensive assessment, two distinct methodologies were employed: the MTT assay was used to measure metabolic activity [64], and flow cytometry was used to assess membrane integrity [65]. The MTT assay was further performed at two different MOIs. Our results revealed that rVSV-dM51-mIL12-mGMCSF does not demonstrate a superior capacity to lyse glioblastoma cells in vitro in comparison to the control virus in either cell line. Furthermore, the control virus demonstrated an amplified oncolytic effect, as indicated by the findings of the flow cytometry analysis. This can be attributed to the increased genome size and metabolic burden associated with the expression of dual cytokines (mIL-12 and mGM-CSF). Owing to its larger genome size, rVSV-dM51-mIL12-mGMCSF is expected to replicate more slowly than the compact rVSV-dM51-GFP, consistent with findings in similarly engineered VSV vectors. These findings are consistent with previous data involving other cancer cell lines [40], suggesting that the enhanced in vivo efficacy is not merely the result of direct viral oncolytic activity. Notably, the VSV platform exhibits extreme potency and rapid kinetics in vitro. Therefore, extending the monitoring period to longer time points was impractical from a biological perspective; extended incubation leads to complete confluency and nutrient depletion in control wells, while near-complete oncolysis is achieved in treated groups, resulting in virtually no viable cells remaining and saturating the cytotoxicity curves.

Rather, these data imply that, even within the immunodeficient environment of athymic nude mice, the expression of mIL-12 and mGM-CSF contributes to tumour control. Although these mice exhibit a profound impairment of adaptive T-cell immunity, they retain functional innate immune components, including natural killer (NK) cells, macrophages, and neutrophils [66]. These results suggest that the residual innate immune components in athymic nude mice are responsive to cytokine-mediated modulation. We therefore hypothesise that local expression of mIL-12 and mGM-CSF may recruit and activate residual innate immune cells, thereby augmenting the direct oncolytic effect of the VSV, despite the absence of a mature T-cell response. This interpretation is consistent with the in vivo results, which demonstrate that the control virus was also ineffective in achieving substantial tumour suppression compared to the negative control. Interestingly, the control virus exhibited higher oncolytic activity in vitro than the cytokine-armed virus. However, this reduction in viral activity was effectively compensated for in vivo by the local IT expression of mIL-12 and mGM-CSF. It should be noted that this IHC analysis in this study focused primarily on the dual-payload rVSV-dM51-mIL12-mGMCSF virus and the vehicle control to validate transgene expression and its direct antiproliferative effects. The rVSV-dM51-GFP control group was omitted from the IHC panel, which is a minor limitation of the current study. Future studies should include this group to enable a more detailed and direct comparison of viral kinetics and cytokine-mediated responses in vivo.

The proposed mechanism also needs to be validated experimentally.

**Figure 6:**
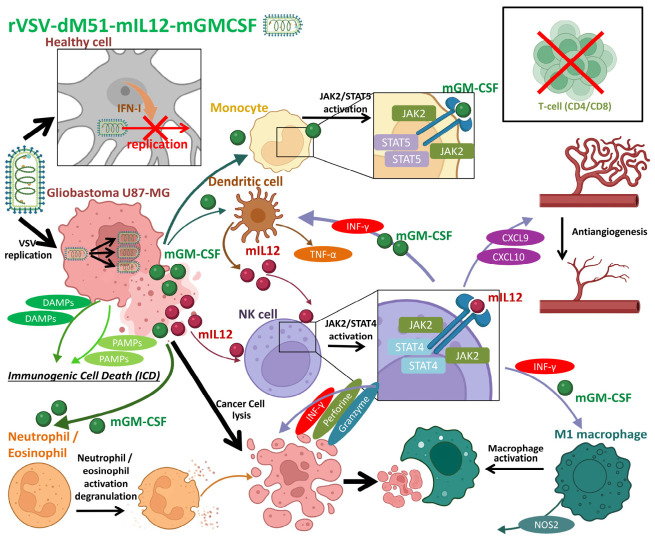
Proposed conceptual model of the synergistic action of rVSV-dM51-mIL12-mGMCSF on tumour cells within a T-cell-deficient tumour microenvironment based on our experimental outcomes and current literature.

Following intratumoural delivery, rVSV preferentially infects U-87 MG cells, leveraging their impaired type I IFN responses to achieve robust viral replication [67]. This selectivity is also increased by the ΔM51 mutation in the VSV matrix protein (VSV M), which reduces the virus’s ability to prevent the export of host mRNA from the nucleus. This process enables healthy cells to sustain protective IFN signalling [24,68] whilst simultaneously promoting immunogenic cell death (ICD) within malignant cells. As an oncolytic agent, VSV has been shown to stimulate both innate and adaptive immune responses. In particular, the lysis of malignant cells by VSV leads to the release of DAMPs and PAMPs. It is well established that innate immune cells, including dendritic cells (DCs) and NK cells, can recognise these patterns and thus induce antitumour immunity. However, while VSV-mediated oncolysis has been shown to trigger the release of DAMPs and PAMPs [2,24], according to our results, this alone is insufficient to overcome the highly immunosuppressive glioblastoma microenvironment, particularly in athymic nude mice, which lack T-cell responses. This is because rVSV-dM51-GFP is ineffective at achieving substantial tumour suppression compared to the negative control. In this context, we hypothesise that integrating IL-12 and GM-CSF provides the necessary co-stimulatory support to actively recruit and reprogramme residual innate immune components. In the absence of T cells, the activation of NK cells becomes the primary cytotoxic effector mechanism, with the capacity to directly target both virus-infected and bystander tumour cells. The virally encoded mouse IL-12, which is secreted by infected tumour cells, binds to IL-12Rβ1/β2 receptors on DCs and NK cells. This activates the JAK2/TYK2–STAT4 signalling pathway. This process has been shown to induce high levels of IFN-γ production, which: (1) enhances NK cell cytotoxicity via the perforin/granzyme pathway; (2) polarises macrophages towards an M1 phenotype with increased nitric oxide synthase activity [69]; and (3) stimulates the production of the chemokines CXCL9 and CXCL10, thereby inhibiting tumour angiogenesis [70] (Figure 6).

Additionally, GM-CSF production from the transgene activates CD116/CD131 receptors on myeloid progenitors and circulating monocytes, thereby activating JAK2–STAT5 signalling. This process induces: (1) differentiation of dendritic cells from myeloid precursors, enhancing antigen processing and IL-12 and TNF-α secretion; (2) recruitment and activation of macrophages with enhanced phagocytic capacity and inflammatory mediator release; and (3) mobilisation of neutrophils and eosinophils, which contribute to tumour cytotoxicity through degranulation and the release of reactive oxygen species and proteolytic enzymes [71,72]. In immunocompetent hosts, the full immunological effect of GM-CSF involves T-cell priming. In athymic nude mice, however, GM-CSF synergises with IL-12-driven IFN-γ responses, increasing NK cell and macrophage activity. The presence of IL-12 and GM-CSF generates a positive feedback loop: GM-CSF-expanded dendritic cells secrete additional IL-12 to reinforce NK cell activation, and IL-12-activated NK cells release IFN-γ to further stimulate the pro-inflammatory activity of macrophages and dendritic cells. Furthermore, both cytokines enhance the recruitment of innate immune effectors to the TME, thereby maintaining inflammatory pressure against the tumour. The combined effects of IL-12-induced chemokines (CXCL9/CXCL10) and myeloid-derived angiostatic mediators result in reduced microvessel density, impaired nutrient supply and localised hypoxia within the tumour mass [70,73,74]. We therefore propose that this integrated innate immune network compensated for the absence of adaptive immunity in athymic nude mice, thereby enabling sustained tumour suppression.

## 5. Conclusions

This study demonstrates the antitumor activity of rVSV-dM51-mIL12-mGMCSF in the inhibition of tumour progression in a U-87 MG glioblastoma model. It was established that the therapeutic superiority of this dual-cytokine-armed virus is not driven by enhanced direct oncolysis, as it exhibited slower in vitro cytotoxicity compared to the parental control virus. Rather, its potent in vivo antitumour effect is mediated by the localised expression of mIL-12 and mGM-CSF, which successfully reprogram the innate immune landscape and induce angiostatic remodelling of the tumour microenvironment. These findings underscore the notion that, even in T-cell-depleted mice, the activation of innate immune elements can effectively compensate for diminished viral fitness, thereby maintaining tumour inhibition and reducing cell proliferation. The recombinant virus is shown to maintain a critical safety profile in normal cells while overcoming the ‘cold’ immunophenotype of GBM. These results suggest that using this virus could be a promising strategy for bypassing the immunosuppressive barriers of glioblastoma. Although the current study is limited by the absence of single-cytokine-armed control viruses (rVSV-dM51-mIL12 and rVSV-dM51-mGMCSF), these findings provide a robust rationale for the further evaluation of rVSV-dM51-mIL12-mGMCSF in immunocompetent glioblastoma models. Future studies to isolate the specific effects of each cytokine and to determine the full potential of the dual-cytokine-armed virus for inducing long-term adaptive antitumour immunity will be a priority.

## Data Availability

The original data presented in the study are openly available in Zenodo at https://doi.org/10.5281/zenodo.21067719.

## Abbreviations

The following abbreviations are used in this manuscript:

rVSV	Recombinant vesicular stomatitis virus
OV	Oncolytic viruses
TME	Tumour microenvironment
mIL-12	Murine interleukin-12
mGM-CSF	Murine granulocyte-macrophage colony-stimulating factor
GFP	Green fluorescence protein
ATCC	American type culture collection
DMEM	Dulbecco’s Modified Eagle’s Medium
FBS	Foetal bovine serum
TCID50	50% tissue culture infectious dose
DAMPs	Damage-associated molecular patterns
PAMPs	Pathogen-associated molecular patterns
MOI	Multiplicity of infection
NK cells	Natural killer cells
MTT	3-(4,5-dimethylthiazol-2-yl)-2,5-diphenyltetrazolium bromide
IT	Intratumoural
PBS	Phosphate-buffered saline
